# Supplementary material for: Intimate partner violence is a barrier to antiretroviral therapy adherence among HIV-positive women: Evidence from government facilities in Kenya
Source: PLoS One. 2021 Apr 21;16(4):e0249813. doi: 10.1371/journal.pone.0249813 (PMC8059826; doi:10.1371/journal.pone.0249813)
Supplement: S1 Table — (DOCX) [file pone.0249813.s001.docx]

**S1 Table 1.** **Propotional Stratified Sampling based on the number of women on ART and location of clinic**

| HIV Clinic | Area | No. of active women on ART | % share of the sample size* | No. of participants |
| --- | --- | --- | --- | --- |
| Bokoli Sub District Hospital | Rural | 229 | 0.6% | 11 |
| Bumala A Health Center | Rural | 831 | 2.1% | 25 |
| Burnt Forest Sub County Hospital | Rural | 959 | 2.4% | 22 |
| Busia County Referral Hospital | Urban | 3824 | 9.5% | 40 |
| Chulaimbo County Hospital | Urban | 3467 | 8.6% | 37 |
| Iten County Referral Hospital | Urban | 618 | 1.5% | 21 |
| Kitale County Hospital | Urban | 4792 | 11.9% | 51 |
| Moi Teaching and Referral Hospital | Urban | 8101 | 20.1% | 80 |
| Mosoriot Sub County Hospital | Rural | 1502 | 3.7% | 20 |
| Mukhobola Health Center | Rural | 593 | 1.5% | 25 |
| Port Victoria Sub County Hospital | Rural | 1500 | 3.7% | 40 |
| Webuye County Hospital | Urban | 2395 | 5.9% | 36 |
| Total |  |  |  | 408 |
