# Supplementary material for: Intimate partner violence is a barrier to antiretroviral therapy adherence among HIV-positive women: Evidence from government facilities in Kenya
Source: PLoS One. 2021 Apr 21;16(4):e0249813. doi: 10.1371/journal.pone.0249813 (PMC8059826; doi:10.1371/journal.pone.0249813)
Supplement: S2 Table — (DOCX) [file pone.0249813.s002.docx]

**SI Table 2 Multiple regression models of factors associated with ART adherence among women who experienced IPV in the last 12 months**

| **Predictor Variable** |  | **Dependent variable = ART** | | | | | | | | | | | | | | |
| --- | --- | --- | --- | --- | --- | --- | --- | --- | --- | --- | --- | --- | --- | --- | --- | --- |
|  |  | **Model 1** | | |  | **Model 2** | | |  | **Model 3** | | |  | **Model 4** | | |
|  |  | ***AOR*** | ***95% CI*** | ***p*** |  | ***AOR*** | ***95% CI*** | ***p*** |  | ***AOR*** | ***95% CI*** | ***p*** |  | ***AOR*** | ***95% CI*** | ***p*** |
| **Physical IPV** | (None) |  |  |  |  |  |  |  |  |  |  |  |  |  |  |  |
|  | Yes | 0.58 | 0.34 - 0.98 | .044* |  |  |  |  |  |  |  |  |  |  |  |  |
| **Sexual IPV** | (None) |  |  |  |  |  |  |  |  |  |  |  |  |  |  |  |
|  | Yes |  |  |  |  | 0.52 | 0.30 - 0.88 | .016* |  |  |  |  |  |  |  |  |
| **Emotional IPV** | (None) |  |  |  |  |  |  |  |  |  |  |  |  |  |  |  |
|  | Yes |  |  |  |  |  |  |  |  | 0.53 | 0.31 - 0.90 | .021* |  |  |  |  |
| **Controlling Behaviour** | (None) |  |  |  |  |  |  |  |  |  |  |  |  |  |  |  |
|  | Yes |  |  |  |  |  |  |  |  |  |  |  |  | 0.56 | 0.33 - 0.93 | .026* |
| **Age** |  | 1.01 | 0.99 - 1.05 | .196 |  | 1.02 | 0.99 - 1.05 | .133 |  | 1.02 | 0.99 - 1.05 | .130 |  | 1.01 | 0.99 - 1.05 | .191 |
| **TARV** |  | 1.00 | 0.99 - 1.00 | .506 |  | 1.00 | 0.99 - 1.00 | .523 |  | 1.00 | 0.99 - 1.00 | .429 |  | 1.00 | 0.99 - 1.00 | .662 |
| **Education** | (None) |  |  |  |  |  |  |  |  |  |  |  |  |  |  |  |
|  | Primary | 2.18 | 0.78 - 6.09 | .129 |  | 2.23 | 0.81 - 6.17 | .116 |  | 2.28 | 0.82 - 6.38 | .112 |  | 2.00 | 0.73 - 5.51 | .172 |
|  | Secondary | 1.29 | 0.44 - 3.79 | .632 |  | 1.38 | 0.47 - 4.04 | .542 |  | 1.30 | 0.44 - 3.83 | .621 |  | 1.24 | 0.42 - 3.60 | .686 |
|  | Tertiary | 4.59 | 1.01 - 22.39 | .050. |  | 5.11 | 1.13 - 24.91 | .037* |  | 4.83 | 1.05 - 23.84 | .046* |  | 4.63 | 1.02 - 22.57 | .050. |
| **Marital Status** | (In a relationship) |  |  |  |  |  |  |  |  |  |  |  |  |  |  |  |
|  | Monogamous marriage | 1.69 | 0.73 - 3.91 | .213 |  | 1.42 | 0.61 - 3.27 | .409 |  | 1.65 | 0.71 - 3.79 | .238 |  | 1.63 | 0.70 - 3.75 | .247 |
|  | Polygamous marriage | 0.95 | 0.38 - 2.38 | .923 |  | 0.83 | 0.33 - 2.06 | .689 |  | 0.96 | 0.38 - 2.40 | .938 |  | 0.94 | 0.37 - 2.35 | .904 |
| **Area** | (Rural) |  |  |  |  |  |  |  |  |  |  |  |  |  |  |  |
|  | Urban | 1.26 | 0.78 - 2.05 | .335 |  | 1.27 | 0.78 - 2.07 | .316 |  | 1.22 | 0.75 - 1.98 | .420 |  | 1.27 | 0.78 - 2.06 | .319 |
| **Partner`s Alcohol Consumption** | (None) |  |  |  |  |  |  |  |  |  |  |  |  |  |  |  |
|  | Sometimes | 0.69 | 0.40 - 1.17 | .170 |  | 0.66 | 0.39 - 1.13 | .132 |  | 0.72 | 0.42 - 1.22 | .228 |  | 0.67 | 0.39 - 1.13 | .139 |
|  | Often | 1.57 | 0.77 - 3.26 | .213 |  | 1.51 | 0.751 - 3.13 | .250 |  | 1.62 | 0.80 - 3.37 | .181 |  | 1.43 | 0.71 - 2.94 | .315 |
| **Partner’s HIV Status** | (Negative) |  |  |  |  |  |  |  |  |  |  |  |  |  |  |  |
|  | Positive | 1.68 | 1.02 - 2.77 | .039* |  | 1.64 | 1.00 - 2.71 | .049* |  | 1.56 | 0.95 - 2.58 | .076 |  | 1.60 | 0.97 - 2.63 | .061 |
| **Supporting partner** | (No) |  |  |  |  |  |  |  |  |  |  |  |  |  |  |  |
|  | Yes | 1.26 | 0.76 - 2.09 | .362 |  | 1.24 | 0.75 - 2.07 | .389 |  | 1.21 | 0.73 - 2.01 | .448 |  | 1.28 | 0.77 - 2.12 | .330 |
| **Woman is Violent** | (No) |  |  |  |  |  |  |  |  |  |  |  |  |  |  |  |
|  | Yes | 0.48 | 0.16 - 1.41 | .185 |  | 0.48 | 0.16 - 1.40 | .182 |  | 0.51 | 0.17 - 1.47 | .213 |  | 0.49 | 0.16 - 1.45 | .200 |
|  | Fights back | 0.47 | 0.22 - 0.98 | .046* |  | 0.46 | 0.21 - 0.98 | .046* |  | 0.47 | 0.22 - 1.00 | .051. |  | 0.44 | 0.20 - 0.92 | .030* |
| R^2^ _Hosmer & Lemeshow_ |  | .10 | | |  | .11 | | |  | .11 | | |  | .11 | | |
| R^2^ _Cox & Snell_ |  | .13 | | |  | .13 | | |  | .13 | | |  | .13 | | |
| R^2^_Nagelkerke_ |  | .17 | | |  | .18 | | |  | .18 | | |  | .18 | | |
