# Supplementary material for: Intimate partner violence is a barrier to antiretroviral therapy adherence among HIV-positive women: Evidence from government facilities in Kenya
Source: PLoS One. 2021 Apr 21;16(4):e0249813. doi: 10.1371/journal.pone.0249813 (PMC8059826; doi:10.1371/journal.pone.0249813)
Supplement: S3 Table — (DOCX) [file pone.0249813.s003.docx]

**SI Table 3. Multiple regression models of factors associated with ART adherence including clinics as a predictor variable**

| **Predictor Variable** |  | **Dependent variable = ART** | | | | | | | | | | | | | | |
| --- | --- | --- | --- | --- | --- | --- | --- | --- | --- | --- | --- | --- | --- | --- | --- | --- |
|  |  | **Model 1** | | |  | **Model 2** | | |  | **Model 3** | | |  | **Model 4** | | |
|  |  | ***AOR*** | ***95% CI*** | ***p*** |  | ***AOR*** | ***95% CI*** | ***p*** |  | ***AOR*** | ***95% CI*** | ***p*** |  | ***AOR*** | ***95% CI*** | ***p*** |
| **Physical IPV** | (None) |  |  |  |  |  |  |  |  |  |  |  |  |  |  |  |
|  | Yes | 0.52 | 0.29-0.93 | .027* |  |  |  |  |  |  |  |  |  |  |  |  |
| **Sexual IPV** | (None) |  |  |  |  |  |  |  |  |  |  |  |  |  |  |  |
|  | Yes |  |  |  |  | 0.48 | 0.27 -0.85 | .012 * |  |  |  |  |  |  |  |  |
| **Emotional IPV** | (None) |  |  |  |  |  |  |  |  |  |  |  |  |  |  |  |
|  | Yes |  |  |  |  |  |  |  |  | 0.66 | 0.36-1.21 | .185 |  |  |  |  |
| **Controlling Behaviour** | (None) |  |  |  |  |  |  |  |  |  |  |  |  |  |  |  |
|  | Yes |  |  |  |  |  |  |  |  |  |  |  |  | 0.43 | 0.23- 0.77 | .005** |
| **Age** |  | 1.01 | 0.98-1.05 | .321 |  | 1.02 | 0.99- 1.05 | .268 |  | 1.02 | 0.98-1.05 | .365 |  | 1.01 | 0.98- 1.05 | .472 |
| **TARV** |  | 1.00 | 0.99-1.00 | .219 |  | 1.00 | 0.99- 1.01 | .284 |  | 1.00 | 0.99- 1.00 | .210 |  | 1.00 | 0.99- 1.00 | .418 |
| **Education** | (None) |  |  |  |  |  |  |  |  |  |  |  |  |  |  |  |
|  | Primary | 1.77 | 0.58-5.52 | .316 |  | 1.67 | 0.55- 5.11 | .360 |  | 1.61 | 0.53- 4.93 | .396 |  | 1.52 | 0.50- 4.64 | .457 |
|  | Secondary | 1.13 | 0.34-3.74 | .843 |  | 1.15 | 0.35- 3.81 | .813 |  | 1.05 | 0.32- 3.45 | .931 |  | 1.00 | 0.31- 3.31 | .995 |
|  | Tertiary | 7.71 | 1.41-46.59 | .021* |  | 8.34 | 1.55-49.35 | .015* |  | 6.41 | 1.20-37.49 | 0.033* |  | 6.52 | 1.22-38.45 | .032* |
| **Marital Status** | (In a relationship) |  |  |  |  |  |  |  |  |  |  |  |  |  |  |  |
|  | Monogamous marriage | 2.08 | 0.81-5.39 | .126 |  | 1.57 | 0.62-3.96 | .339 |  | 1.95 | 0.76- 4.98 | .161 |  | 1.70 | 0.67- 4.33 | .263 |
|  | Polygamous marriage | 0.84 | 0.29-2.33 | .730 |  | 0.66 | 0.23- 1.80 | .417 |  | 0.78 | 0.28-2.14 | .625 |  | 0.73 | 0.26-2.01 | .536 |
| **Area** | (Rural) |  |  |  |  |  |  |  |  |  |  |  |  |  |  |  |
|  | Urban | NA | NA | NA |  | NA | NA | NA |  | NA | NA | NA |  | NA | NA | NA |
| **Partner`s Alcohol Consumption** | (None) |  |  |  |  |  |  |  |  |  |  |  |  |  |  |  |
|  | Sometimes | 0.69 | 0.38-1.24 | .215 |  | 0.66 | 0.36-1.18 | .162 |  | 0.71 | 0.39-1.29 | .257 |  | 0.67 | 0.37-1.21 | .180 |
|  | Often | 1.97 | 0.87-4.59 | .105 |  | 1.87 | 0.83-4.33 | .135 |  | 1.96 | 0.87-4.55 | .108 |  | 1.81 | 0.81-4.16 | .153 |
| **Partner’s HIV Status** | (Negative) |  |  |  |  |  |  |  |  |  |  |  |  |  |  |  |
|  | Positive | 1.89 | 1.09-3.32 | .023* |  | 1.76 | 1.03- 3.06 | .039* |  | 1.74 | 1.00- 3.02 | .048* |  | 1.65 | 0.96-2.84 | .068 |
| **Supporting partner** | (No) |  |  |  |  |  |  |  |  |  |  |  |  |  |  |  |
|  | Yes | 1.41 | 0.79-2.51 | .235 |  | 1.36 | 0.78-2.38 | .272 |  | 1.42 | 0.80-2.52 | .228 |  | 1.41 | 0.80-2.47 | .223 |
| **Woman is Violent** | (No) |  |  |  |  |  |  |  |  |  |  |  |  |  |  |  |
|  | Yes | 0.36 | 0.10-1.22 | .103 |  | 0.39 | 0.11-1.32 | .132 |  | 0.36 | 0.10-1.22 | .104 |  | 0.41 | 0.11-1.47 | .173 |
|  | Fights back | 0.57 | 0.25-1.27 | .169 |  | 0.56 | 0.245-1.26 | .164 |  | 0.523 | 0.23-1.18 | .119 |  | 0.55 | 0.24-1.24 | .152 |
| **Clinic** | (MTRH Module 3) |  |  |  |  |  |  |  |  |  |  |  |  |  |  |  |
|  | Burnt Forest Sub-County Hospital | 0.09 | 0.00-0.89 | .068 |  | 0.09 | 0.00-0.88 | .065 |  | 0.08 | 0.00- 0.77 | .051. |  | 0.09 | 0.00- 0.88 | .065 |
|  | Busia County Referral Hospital | 1.56 | 0.45-5.60 | .476 |  | 1.55 | 0.45-5.57 | .484 |  | 1.46 | 0.42-5.23 | .548 |  | 1.59 | 0.47-5.70 | .460 |
|  | Chulaimbo County Hospital | 1.72 | 0.56-5.45 | .344 |  | 1.92 | 0.62- 6.03 | .260 |  | 1.61 | 0.52-5.03 | .410 |  | 1.58 | 0.51-4.94 | .428 |
|  | Iten County Referral Hospital | 1.26 | 0.33-4.99 | .736 |  | 1.27 | 0.34-5.02 | .722 |  | 1.20 | 0.31-4.80 | .789 |  | 1.29 | 0.35-5.11 | .701 |
|  | Kitale County Hospital | 3.49 | 1.09-11.66 | .037* |  | 4.05 | 1.25-13.80 | .021* |  | 3.11 | 0.97-10.29 | .057 |  | 3.72 | 1.14-12.71 | .031* |
|  | Mosoriot Sub County Hospital | 1.82 | 0.44-8.30 | .417 |  | 2.01 | 0.47-9.34 | .353 |  | 1.75 | 0.42-8.03 | .453 |  | 2.06 | 0.49-9.26 | .327 |
|  | MTRH Module 1 | 0.22 | 0.07-0.65 | .007** |  | 0.26 | 0.08-0.75 | .015* |  | 0.21 | 0.06-0.63 | .006** |  | 0.30 | 0.09-0.86 | .028* |
|  | Mukhobola Health Center | 0.31 | 0.08-1.09 | .072 |  | 0.37 | 0.09-1.34 | .136 |  | 0.28 | 0.07-0.98 | .052 |  | 0.24 | 0.06-0.83 | .028* |
|  | Port Victoria Sub County Hospital | 0.63 | 0.20-1.93 | .422 |  | 0.74 | 0.23-2.29 | .608 |  | 0.69 | 0.22-2.11 | .518 |  | 0.63 | 0.19-1.91 | .414 |
|  | Webuye County Hospital | 0.95 | 0.28-3.22 | .946 |  | 1.17 | 0.34-3.94 | .803 |  | 0.78 | 0.24- 2.56 | .684 |  | 0.88 | 0.27-2.85 | .825 |
|  | Bumala A Health Center | 11.52 | 2.33-89.34 | .006** |  | 12.10 | 2.41-94.94 | .005** |  | 10.59 | 2.17- 81.04 | .007** |  | 14.65 | 2.91- 114.45 | .002** |
|  | Bokoli Sub District Hospital | 0.50 | 0.08-2.65 | .420 |  | 0.43 | 0.07-2.18 | .313 |  | 0.39 | 0.07-2.01 | .269 |  | 0.51 | 0.09-2.70 | .435 |
| R^2^ _Hosmer & Lemeshow_ |  | .23 | | |  | .23 | | |  | .22 | | |  | .23 | | |
| R^2^ _Cox & Snell_ |  | .26 | | |  | .26 | | |  | .25 | | |  | .26 | | |
| R^2^_Nagelkerke_ |  | .35 | | |  | .35 | | |  | .34 | | |  | .36 | | |
