# Supplementary material for: Intimate partner violence is a barrier to antiretroviral therapy adherence among HIV-positive women: Evidence from government facilities in Kenya
Source: PLoS One. 2021 Apr 21;16(4):e0249813. doi: 10.1371/journal.pone.0249813 (PMC8059826; doi:10.1371/journal.pone.0249813)
Supplement: Attachment — Submitted filename: Plos_adherence.pdf [file pone.0249813.s005.pdf]

Comments on ”Intimate partner violence is a barrier to antiretroviral therapy adherence among HIV-positive women: Evidence from government facilities in Kenya”

The current version of the paper is well written, and the description of methods and results is easy to follow. As far as I can see, the authors have addressed the comments by the previous reviewers satisfactorily. However, there are still some issues that should be addressed.

The authors correctly use the word association throughout the paper. However, the conclusion (in the abstract and elsewhere) that “ART programs should consider incorporating basic IPV interventions into regular clinic services and using healthcare providers as information and navigation points for women exposed to IP” does not follow from the findings. Such a conclusion should be based on evidence of a casual effect of IPV on ART adherence. An association does not have to be due to the impact of IPV on adherence. For example, women who end up with violent men might be different from others in ways not captured by the control variables, making them care less about their health. Moreover, violent men might have characteristics that influence their partners’ adherence. One example is that they are likely to be less faithful and less caring. Thus, a program that reduces IPV might not improve adherence. Although it seems reasonable that IPV actually has a causal effect on adherence, the conclusion drawn from the study should be modified.

A related issue is the consequences of IPV on HIV-infections. The authors describe these in the first two paragraphs of the paper and refer to several papers. However, they miss some important references and the fact that recent evidence indicates that there might not be a causal effect of IPV on HIV-infections among women in Sub-Saharan Africa. Women who live with violent men are primarily infected because violent men are more likely than others to be HIV-infected. See for example, Durevall and Lindskog (2015) and Heise and McGrory (2016).

A drawback of the study is that women who have not entered an ART program or have dropped out are not included in the sample. This should be made clear early on, particularly since it is likely to create a bias that weakens the association. It should also be commented on in Limitations. Now it is mentioned in Conclusion, which is odd.

I miss a description of why there might be an association between IPV and adherence in Introduction.

The authors use self-reported lifetime IPV restricted to the current partner. Since they first write that they use the DHS IPV module and then (correctly) state that it measures IPV by current and former partners (page 6-7), the claim that the authors focus on lifetime IPV of the ongoing relationship is confusing. Either the claim is wrong or the authors have modified the DHS IPV module. If they have modified it, this should be clearly stated.

Since data on IPV during the last 12 months are available, which in my view would have been more suitable for the study than lifetime IPV, the authors should at least comment on how the results are altered if they are used.

The authors write as if unadjusted results are reported, see p. 11 lin3-4. However, they are not reported.

What does marital status, monogamy, polygamy in Table 1 mean? The classification is unclear.

Typo, p.19, line 6. on should be of.

References

Durevall, D., & Lindskog, A. (2015). Intimate partner violence and HIV infection in sub-Saharan Africa. World Development, 72, 27-42.

Heise, L., & McGrory, E. (2016). Violence against women and girls and HIV: Report on a high level consultation on the evidence and its implications, 12–14 May, 2015. *Greentree Estate: STRIVE Research Consortium, London School of Hygiene and Tropical Medicine*.
