# Supplementary material for: Intimate partner violence is a barrier to antiretroviral therapy adherence among HIV-positive women: Evidence from government facilities in Kenya
Source: PLoS One. 2021 Apr 21;16(4):e0249813. doi: 10.1371/journal.pone.0249813 (PMC8059826; doi:10.1371/journal.pone.0249813)
Supplement: Attachment — Submitted filename: Response to Reviewers.docx [file pone.0249813.s004.docx]

Bornice C. Biomndo,

Charité - Universitätsmedizin Berlin,

Reinickendorfer Straße 61 D-13347,

Berlin, Germany

31.10.2020

To the Editor-in-Chief,

Dear Dr. Munro-Kramer,

We appreciate the time you and the reviewers took to read through our manuscript and provide constructive criticism and comments. We have considered all of the feedback and used it to develop an improved version of the manuscript, “Intimate partner violence is a barrier to antiretroviral therapy adherence among HIV-positive women: Evidence from government facilities in Kenya”. After incorporating the helpful comments into our revision, we believe the quality of the manuscript has improved immensely. Therefore, we would like to resubmit it for consideration for publication in PLOS ONE.

We have included the comments on the following pages of this letter and provided a point-by-point response that details how we addressed each comment, which revisions were made, and where they can be found. The responses are italicized to distinguish them from the comments. Additionally, we have uploaded our data set to Zenodo and it is available under the following DOI: 10.5281/zenodo.4135394.

Sincerely,

Bornice Biomndo,

Charité - Universitätsmedizin Berlin

**Editor’s comments**

1) There are numerous grammatical errors and missing words throughout the manuscript that make it

difficult to understand what the authors are saying at times. Please consider enlisting the help of a copy editor prior to resubmission.

*Thank you for the feedback. Reviewers #1 and #2 also commented on the grammatical errors and typos. We have therefore* *changed the title to read ‘Intimate partner violence is a barrier to antiretroviral therapy adherence among HIV-positive women: Evidence from government facilities in Kenya’. Furthermore, we have rectified the typos and grammatical errors within the manuscript and hired a professional copy editor to review the final draft.*

2) Please ensure consistency of terminology, specifically related to the key concepts in the manuscript

(e.g., intimate partner violence (IPV) including physical IPV, sexual IPV, and emotional IPV; antiretroviral therapy). Define terms that may not be well understood (e.g., mixed clinics). Abbreviations should be introduced and then used consistently.

*We thank you for this correction. Reviewers #1 and #2 also commented that the term ‘mixed clinics’ was unclear. Reviewer #2 pointed out that the term ‘IPV’ was loosely and inconsistently used, that consistency was necessary, and that the different types of violence should be specified as physical IPV, emotional IPV, etc. We have revised the manuscript to clearly and consistently identify the forms of violence as IPV and to consistently use the term IPV. We have rewritten the part of the abstract that mentioned ‘mixed clinics’ (Pg. 2 line 28). Within the methods section, we have clarified in Pg. 8 lines 145 and 146 that ‘mixed clinics refer to non-specialized clinics where female and male HIV-positive adults are reviewed and given medication refills.*

3) Citations need to be consistent per PLOS One format (Vancouver Style) and should be used for

each claim that references supporting literature. For example, the introduction cites "IPV is a known risk factor for HIV infection..." without a citation. Also, all tables/figures should be cited in the text.

*Thank you for pointing out this oversight. Reviewers #1 and #2 also commented on citation. Specifically, in the previous manuscript, Reviewer #1 noted that all citations should be in a consistent format and gave the example of Pg. 14 line 241, which was not consistent with the format. Reviewer #2 pointed out that it was inappropriate to claim that IPV is “recognised as a factor behind low uptake and engagement of HIV care” and provide only one citation. Additionally, some sentences (e.g. Pg. 5 lines 58-60) were missing citations. In the revised manuscript we have ensured that all references are appropriately cited (i.e. Pg. 15 line 259). We have added six citations to support the introductory statement that ‘Intimate Partner Violence (IPV) is recognised as a factor behind low uptake and engagement of HIV management services’, and added citations in Pg. 4 lines 58 and 59 for the studies we referred to. Moreover, regarding the claim that ‘IPV is a risk factor for HIV infection among women....’ (Pg. 4 line 59), we have added two citations and separately referenced lines 60-62, which we had previously erroneously combined into one citation.*

*For the tables and figures, we have included the traditional Table 1 as advised and created a new table that we believe gives a concise and clear summary of the logistic regression analysis results. We have also confirmed that all relevant tables are mentioned in the texts.*

4) The methods section needs additional details about measures and procedures including:

(a) How were the 12 clinics selected (e.g., was it a random sample)?

*Within the study design and setting section, we have added a sentence explaining that the 12 clinics were selected based on the number of active HIV-positive women on ART they, and also to represent the different counties (regions) in western Kenya. Consequently, we included large clinics with high numbers of women on ART in the different counties. Additionally, smaller clinics were selected to have representation from less populous communities. We have rewritten the information in the methods section (Pg.5 lines 93–97) to clarify this.*

(b) For inclusion criteria, what were the parameters around "in an intimate relationship"? Did this have to be at the time of the study? At any point in their life?

(c) Were the IPV measurements for lifetime? If so, why was this time frame used when ART adherence focused on the last 30 days?

*We appreciate the detailed feedback and we acknowledge that the study’s definition and measurement of IPV was somewhat unclear in the previous manuscript. Reviewer 1# also commented that, while adherence was measured over the past 30 days, the description suggested that IPV was a lifetime measure. Reviewer #1 stated that we should explain why IPV was not measured in the same timeframe as adherence (or at least more recently, such as the past 3 months). Since these comments are related, we decided to combine them and offer a comprehensive response*.

*Our study focuses on the current relationship and the occurrence of IPV within the relationship’s lifetime. We have clarified this by including within the data collection section the description of the IPV measurement and a sentence defining an intimate partner as someone to whom the woman was currently married (whether in a monogamous or polygamous marriage) or a person with whom she was in a romantic relationship (Pg. 6 Lines 118 and 119). We have also included the words ‘current intimate partner’ and ‘current relationship’ in the abstract, results, and discussion sections whenever we refer to the partner or relationship*.

*We chose to use a relationship-specific timeframe for IPV exposure and ART adherence in the 30 days prior to the survey, because our interest was investigating whether living in an environment where IPV occurs (whether past or ongoing) affects a woman’s ability to adhere to treatment. We hypothesise that there is a potential link. Our reasoning is that, although a specific IPV incident may not affect medication adherence, the impact of living in such a setting is what influences the woman’s ability to maintain optimal ART adherence.*

(d) Is the "recruiter" a trained study staff member or their healthcare provider? Did they have resources available for someone who was actively experiencing IPV and asked for resources? Were participants excluded if there was a safety concern? Were the interviews conducted in private? Were they conducted with paper surveys? Was there an incentive?

*Reviewer #1 also asked whether the women were offered any compensation for participating.* *We have added more information on the recruiters, the survey administration process, and the compensation within the procedure section. The ‘recruiters’ were the women’s regular healthcare providers – i.e. clinical officers and nurses who provide and supervise ART at the clinics. The healthcare providers were trained on the aims of the study, the recruitment process, and questionnaire administration. A list of in-house resources (social workers and, where available, a legal aid office) and external resources (government authorities, non-governmental organisations in the area that work in gender-based violence prevention/women’s empowerment) was compiled and offered to women who reported exposure to IPV.*

*The survey was conducted right after the woman’s routine clinical check-up; therefore, it took place in the same examination room. Although this was very convenient and practical, unfortunately, it meant that if the examination room was shared, the healthcare providers had to ensure some form of privacy when administering the questionnaire. They did so either by asking their fellow healthcare provider to temporarily leave the room or by creating physical distance by moving their desks or dividing the room with a curtain. If the woman was accompanied by another adult or a child who was old enough to understand the conversation, the person/child was politely asked to leave the room before the questionnaire was administered. Women who participated in the survey were compensated for their time and effort in form of cash, which was referred to as ‘transport money’. This was only mentioned and given after the woman completed the questionnaire. This information has been added within the procedure section.*

*In the data collection section, we have added a sentence clarifying that the questionnaire was in paper form (Pg. 7 lines 142 and 143).*

(e) How many participants declined participation? What was the reasoning?

*Thank you for raising this question. In the discussion section, we have added information stating that, despite anticipating some hesitance from the women, all of the healthcare providers reported that the women they approached willingly and openly discussed their IPV experience. We have also included the reasons that we think the women were receptive to the survey (Pg. 14 Lines 244 -248). Although some healthcare providers did require more time to recruit women, this was due to not having enough women who fit the criteria, rather than the women declining to participate.*

5) The results section should be streamlined. First of all, a descriptive/demographic table of the sample

is needed. Second, the current tables contain a large amount of information. The authors should consider how to condense this information in an easily digestible way.

*Thank you for this feedback. We have added Table 1, which contains descriptive information about the sample. The previous tables were certainly large and information-heavy. Therefore, we have developed a single table (Table 2) that combines the previous four models in a concise way that is easier to follow. We decided not to report the results of the single regression analyses within an extra table; instead, we focus on the full regression models, which are more informative.*

*Within Table 2, Pseudo R^2^ values are reported for transparency even though the aim of our model is to explore the association between the independent variables and the dependent variable, and not to predict the dependent variable [**1**,* *2**]. Nevertheless, the arguably low Pseudo R^2^ values were not surprising to us since we expected that there are other variables that affect ART that were not measured in our survey [**3**].*

6) As noted by Reviewer #1, the conclusion could benefit from a future research or future directions

section.

*Reviewer #1 commented that it would be beneficial to provide a ‘Future Research’ or ‘Future Practices’ section that discusses next steps. The Conclusion should briefly summarise the study results and implications.* *We agreed that the discussion and conclusion could be improved and have rewritten them to expound more on the implications of our study; we have specified the practical steps that can be taken and suggested future research. Specifically, we have added a ‘Future practices’ segment within the discussion section (Pg. 18 line 351) that expounds on the practicality of integrating basic IPV screening and counselling within HIV clinics and the role that healthcare providers can play in this regard. We also indicate the need for strategies at the societal level to create awareness on the negative effects of IPV, the laws that protect against it, and the existing resources that are available for women exposed to IPV. To conclude, we have expressed the need for more evidence on the direct and indirect benefits of integrating IPV screening and counselling for women in healthcare settings. We have called on future researchers to target vulnerable groups (e.g. women who drop out of ART programs) and to produce more evidence on the negative effects of controlling behaviour.*

**Reviewer #1**

Abstract

In the results of the abstract the authors state protective factors but it is unclear if they are protective factors regarding increased ART adherence or IPV.

*Noted. We have rewritten the sentence to make it clear that the protective factors regard increased ART adherence (Pg. 2 line 39).*

Introduction:
Overall: The authors state a “gap in the literature” but then cite several studies. It seems that the authors should report conflicting results in the literature rather than a gap. Given the specific populations previous studies focused on, this study adds to the literature by recruiting from multiple types of clinics across locations. I believe this should be further emphasized in the introduction to provide additional rationale of the importance of this study.

*This is true. Our intention was to emphasise that the studies that have aimed to fill this gap in the literature by focusing on specific subsets of women have produced contrasting and inconclusive evidence. Therefore, our study aims to contribute by adding evidence from a broad group of women. We now realise that area was not well-written in the previous manuscript. We have revised parts of the introduction section to make this point more clearly (Pg. 5 lines 74-82).*

Specific:
*The authors state that most studies are conducted in specific regions/cities in Africa, which suggested that the present study would be conducted across sub-Saharan Africa. Discussion on the limitations of location-specific studies should be limited since this study is conducted in Kenya and not across sub-Saharan Africa.

*By mentioning specific locations in our paper, our intention was to highlight that there are studies from other sub-Saharan African countries which may be comparable to our setting and population in Kenya. We did not intend to imply that the study location could be a limiting factor or that our study was conducted in several locations. Any discussion or comparison to a specific study has focused on the sample population (e.g. pregnant and postpartum women, transactional sex workers) or the method of measuring ART adherence (cumulative ART adherence* *and cut-off points*). *However, we now see how highlighting specific locations could have a different effect than we anticipated; therefore, we have limited this in the revised manuscript.*

Methods:
Overall: I have some concerns and questions regarding the measures and data analysis.
Specific:
*If IPV influences ART adherence, why were women included only if they had been on ART for at least 6 months? Is it possible that women may not be on ART in the past 6 months because of IPV?

*We realized that the wording we used to explain this criteria was misleading, and we have rewritten it in Pg.6 lines 100–103. The six-month limit refers to the length of time since initiation of ART – not to the length of time that the women had actively been on ART prior to the survey. Therefore, it did not matter whether or for how long the woman had been off ART prior to the study. They were included as long as the onset of ART was more than six months prior to the survey. Our outcome of interest was ART adherence. Because we hypothesise that some factors affecting adherence are related to the initiation stages of ART, we tried to minimise these factors by capturing women who may have been accustomed to the ART routine.*

*Was there any missing data? If so, how was it accounted for during analyses? Analyses that included multiple locations often use a nested model to account for differences. Did the authors include clinics in analyses?

*We have added a brief paragraph in the data analysis section ( from Pg. 9 line 182) explaining that a missing value analysis was performed and none of the variables had more than 5% missing values; we also did not find any systematic relationships between the missing values. Regarding the nested structure of our data, we have added on Pg. 9 line 190- 193 that we decided against a multilevel modelling procedure because, with as few as 12 clusters, fixed-effect estimates associated with level-2 predictors could have been severely biased.*

Results:
Overall: The tables are somewhat difficult to follow and incorporate a lot of information. Since there are 4 very large tables, the authors should consider a more succinct manor in presenting results.
Specific:
*Figure 1 is not mentioned in the manuscript and it should be referred to as a table rather than a figure

*A demographics table would be helpful, especially if the authors minimize or combine tables 1 – 4.
*Thank you for this feedback. We have made major revisions to the tables and figures, as suggested. We developed Table 1, which contains demographic information on the participants. We have combined the previous tables (Tables 1–4) into a single table (Table 2).*

*We removed the table containing information on the clinics (previously erroneously identified as Figure 1) and offered it as extra supplementary information, since it does not directly relate to the results and only serves as extra information. Additionally, we deleted Figure 2 since the information that was presented is now available in Table 1 and within the text in the results section.*

*It is unclear what ~ refers to in the tables.

*Noted. We have deleted the symbol since we realised that the information was clear without it.*

*Many of the variables listed in the tables are not described in the Method/Measures section.
*We apologise for this oversight. On Pg. 7 line 139–142 we have included all of the variables that are later presented in the tables*

**Reviewer #2**

After careful consideration and review of manuscript number PONE-D-20-21450 entitled, “Intimate partner violence a barrier to antiretroviral therapy adherence among HIV positive women: Evidence from government facilities in Kenya”, it is my recommendation for the authors to submit a major revision of the paper. Details are provided below:

Abstract
1. The introduction section is confusing because the authors note that studies on key populations have produced divergent evidence regarding “this association” but it is unclear what “this association” references…intimate partner violence and poor medication adherence or intimate partner violence and virological failure?

*Noted. By ‘this association’ we were referring to IPV’s link to adverse clinical outcomes. However, since our paper focuses on poor ART adherence, we have decided to rewrite the statement to specify the association between IPV and poor ART adherence (Pg. 2 line 23 -25).*

2. (This comment has already been responded to under Editor’s comment #2)

3. The study design and procedures are unclear in the methods section. I recommend conveying the analysis in a brief, succinct manner to allow room for a sentence or two on the study design and procedures.

*Noted. We have rewritten the section to read ‘We sampled 408 HIV-positive women receiving free ART from different types of HIV clinics at government health facilities, assessing for IPV exposure by a current partner, ART adherence rate, and other factors that affect ART adherence (e.g. education, disclosure). ART adherence rates were measured using the Visual Analogue Scale (VAS); responses were dichotomised at a ≥95% cut-off. Multiple logistic regression models assessed the association between the independent variables and ART adherence*.

4. The study implications are vague. There must be more recommendations than merely integrating IPV screening and counselling services within HIV clinics.

*Thank you for highlighting this. We have revised the conclusion section to read* ‘*IPV is associated with suboptimal ART adherence rates among a broad group of HIV-positive women. ART programs should consider incorporating basic IPV interventions into regular clinic services and using healthcare providers as information and navigation points for women exposed to IPV.’*

Introduction
1. Although there are no overall IPV prevalence estimates available from sub-Saharan Africa, there have been numerous studies conducted from which prevalence estimates can be cited, within a range.

*Thank you for this suggestion. We were able to find some estimates, which we have included in Pg. 4 lines 52 and 53.*

2. and 3. (These comments on citation have already been addressed under the Editor’s comment #3)

4. (This comment on typos and grammatical errors has been addressed under Editor’s comment #1)

5. Pg. 5, lines 81-83 presents a sentence that is awkwardly worded; it is difficult to understand the purpose and meaning of the sentence.

*Noted. We realise our mistake and have rewritten the sentence to read ‘So far, the evidence from sub-Saharan Africa has been based on research among key populations, such as transactional sex workers and women at Prevention of Mother-to-Child Transmission (PMTCT) clinics (Pg. 5 lines 77–79).*

6. In one area, “partner violence” is used and in other areas “intimate partner violence” is used. Is this supposed to represent a distinction?

*No, the intent was not to make any distinction. We acknowledge this mistake and have corrected it by consistently mentioning IPV whenever we talk about violence.*

7. The claim is made that “there needs to be an expansive approach that encompasses the diverse population of HIV positive women” but it is unclear what this approach actually is and how this approach serves as the foundation of the paper.

*Thank you for pointing this out. To clarify what the approach is, we have revised this claim on Pg. 5 line 79–82 to read ‘However, to determine whether IPV exerts an overarching influence on ART adherence, the association between both variables must be explored among a broader sample of HIV-positive women with diverse socioeconomic characteristics who are receiving the available standard ART from different types of HIV clinics.’*

Methods
8. For consistency, once ART has been defined, it is necessary to utilize the acronym throughout the paper. In the methods section, this was not the case.

*Noted. We have revised the manuscript and ensured that the abbreviation is used consistently throughout.*
9. The eligibility criteria does not include participants self-reporting as women; was this not an inclusion criteria?

*The women were recruited from patient lists which are developed from the official medical records. The patient lists are a list of the clients who have clinic appointments on that day. The recruiters used the patient lists to randomly select participants who, based on their official medical records which the clients filled in during their first clinic encounter, are categorised as ‘female’. Since this categorisation was predetermined by how the participant self-identifies in their official medical records, we did not see the need to consider it as an extra criteria. Nevertheless, in the questionnaire, we were conscious to use the term ’partner’ when gathering information about the relationship with the intimate partner, and ‘husband/partner’ when measuring exposure to IPV.*

10. The national IPV prevalence rate was mentioned in the methods section but not included in the introduction section.

*Thank you for highlighting this omission. We have included the national IPV rate in the introduction section (Pg. 4 lines 55 and 56).*

11. (This comment on inconsistent use of the term IPV has been addressed under Editor’s comment #2)
12. (This comment on mixed clinics has been addressed under Editor’s comment #2)

13. More details are needed regarding the administration of the survey (e.g., was the location in a private office, computer administered, etc.).

14. How was participation handled for women who were at the clinic with a partner?

*Added. We have added in Pg. 7 lines 142 and 143 a sentence explaining that the questionnaire was in paper form. Furthermore, within the procedure section (from Pg.8 line 155), we have included more information on where and how the questionnaire was administered, as well as the process. Specifically, we have explained that the survey was administered in the regular examination room by the healthcare provider immediately after the routine clinic visit was completed. Although very practical, the challenge with this set-up was that the healthcare provider had to ensure maximum possible privacy. We have also stated that, in cases where the woman was accompanied by another person or a child who was old enough to understand the questions, the person or child was politely asked to leave the room.*

15. It is inappropriate to consider the variables in the analysis as “predictors” because the data is cross-sectional in nature, not longitudinal.

*Noted. We reviewed the manuscript and have now limited the use of the word ‘predictor’ to only describe the relationship between two variables in the process of statistical modelling. This is in line with OECD’s definition of the term ‘prediction’ within statistical contexts[**4**]. When inferring to the real world, we have used the term ‘independent variable’. We regret any previous oversight in this regard.*

Results
16. The traditional “Table 1” is missing. This table typically provides descriptive statistics to describe the sample.

*Added. We have developed Table 1, which contains demographic information for the participants.*

17. Figure 1 is included but never referred to in the text of the manuscript.

*We removed the table containing information on the clinics (previously erroneously identified as Figure 1) and offered it as extra supplementary information since it does not directly relate to the results and only serves as extra information.*

18. (This comment has been responded to under Editor’s comment #2)

19. The table titles are misleading and need to account for both the primary independent variable and dependent variable.

*Thank you for highlighting this. We have corrected this in Tables 1 and 2, which we have developed to summarise the results.*

Discussion
20. The authors need to be clear that the discussion points are centered around “lifetime” IPV rather than just stating “prevalence of IPV” – this should be “prevalence of lifetime IPV”.

*Agreed. We needed to be clearer on the definition of the prevalence of IPV. We have rewritten the subtitle to read ‘Prevalence of IPV within the current relationship’ on Pg. 10 line 199 and Pg.14 line 236.*

21. The authors make an incorrect declarative statement (pg. 15, line 255). The study findings can only corroborate studies that document an association between IPV and poor adherence to ARV medication, not the association between IPV and poor uptake of ARV medication.

*This is correct and we regret this error. We have corrected the statement to read that the finding corroborates studies that link IPV to poor adherence to ARV therapy among women (Pg. 15 line 276 and 277).*

22. Pg. 16, line 287-290 – This sentence is grammatically incorrect.

*Noted. After reassessing the sentence, we agreed that deleting it would not alter the point we were aiming to make. We deleted the sentence and took the opportunity to restructure the argument about the effects of controlling behaviour (Pg. 17 line 308- 314).*

23. The implications of the study findings are general and do not provide significant details on how the finding could inform practice and policy. Additionally, there is lack of specificity in terms of future research directions resulting from the current study findings.

*We agree that the conclusion could be improved and we have added a section on ‘future practices’ within the discussion section (Pg. 18 line 351), which expounds on the implications of our study and specifies the practical steps that can be taken. Additionally, we completely rewrote the conclusion section to include suggestions on future research (Pg. 19 lines 372).*

24. Limitations should be discussed regarding the time anchor for the measure, lifetime IPV. Recent IPV may have been a better indicator as it relates to impeding ART adherence.

*Thank you for pointing this out. We took the comment into consideration and included in the limitation section (Pg. 18 from line 337) that, although this could have limited our ability to associate IPV to suboptimal ART adherence, the reason for measuring exposure to IPV within the current intimate relationship (regardless of when it occurred) and comparing it to the 30-day self-reported ART adherence was that our aim was to investigate the impact that past or ongoing IPV within the relationship had on ART adherence. Our rationale is that specific IPV incidents may not affect medication adherence; rather, living in an environment in which IPV occurs is what influences the woman’s ability to maintain optimal ART adherence.*

**Journal Requirements**

1)Please ensure that your manuscript meets PLOS ONE's style requirements, including those for file naming.
2)Please state whether you validated the questionnaire prior to testing on study participants. Please provide details regarding the validation group within the methods section.
3)We note that you have indicated that data from this study are available upon request. PLOS only allows data to be available upon request if there are legal or ethical restrictions on sharing data publicly.

*We have uploaded our data set to Zenodo and it is available under the following DOI:* 10.5281/zenodo.4135394*.*

4) Please include a separate caption for each figure in your manuscript.

*We apologise for the oversight. We have included the necessary captions for the tables (we no longer have any figures).*

**References**

1.	Forst J. Regression analysis : An intuitive guide for using and interpreting linear models. Pennsylvania, United States: Statistics By Jim Publishing; 2019.

2.	Editor MB. The Minitab Blog [Internet]. Minitab, editor. State College2013. [cited 2019]. Available from: https://blog.minitab.com/blog/adventures-in-statistics-2/regression-analysis-how-do-i-interpret-r-squared-and-assess-the-goodness-of-fit.

3.	Giselmar A. J. Hemmert LMS, Jan Wieseke, and Heiko Schimmelpfennig. Log-likelihood-based Pseudo-R2 in logistic regression: Deriving sample-sensitive benchmarks. Sociol Method Res. 2018;47(3).

4.	Marriott FHC. A Dictionary of Statistical Terms. 5th ed. Harlow, Essex: Longman Scientific & Technical; 1990. 223 p.
