# Supplementary material for: Intimate partner violence is a barrier to antiretroviral therapy adherence among HIV-positive women: Evidence from government facilities in Kenya
Source: PLoS One. 2021 Apr 21;16(4):e0249813. doi: 10.1371/journal.pone.0249813 (PMC8059826; doi:10.1371/journal.pone.0249813)
Supplement: Attachment — Submitted filename: Response to Reviewers.docx [file pone.0249813.s006.docx]

**Reviewer’s comments**

This manuscript is an interesting foray into an understudied population. The authors should be commended for their work from the previous draft- it seems much improved. However, there are some methodological questions and framing issues that need attention before the manuscript should be considered for publication.

1a) Line 71: Please expound upon the proposed mechanisms in the literature between IPV and HIV management service uptake.

1b) There is a lack of theoretical or conceptual underpinnings here- *why* do the authors proposed IPV being a barrier to ARV uptake? This is not a new concept, so existing literature and concepts just need to be brought into this section and tailored for the study context. The authors briefly mention this in Line 285, but this should be corroboration of an earlier theory-driven hypothesis.

1c) I miss a description of why there might be an association between IPV and adherence in the Introduction.

*Thank you for the comments. We decided to combine the three comments because we believe they stem from the missing conceptual framework which we should have included.*

*Along the HIV care cascade (i.e. HIV infection, diagnosis, linking to care, retention in care, initiation of ART, and achieving viral suppression), there are several points at which IPV is said to interfere with interventions being successful. Different studies have tried to establish and quantify this interference at different steps of the cascade. For our study, we focus on the last step which is achieving viral suppression through ART adherence. However, we briefly introduce the other cascade points within our introduction section as background information on the relationship between IPV and ART adherence.*

*It is true that we failed to include the conceptual framework which could have made this clearer. Therefore in the revised manuscript we included the conceptual framework on ART adherence which guided our research study on Pg. 4 line 70 -73. That is, ‘Alongside treatment regime, provider-patient relationship, clinic setting, and disease characteristics, the conceptual framework on ART adherence identifies sociodemographic drivers as having the potential to influence a patient’s level of medical adherence. Particularly, that there are socio-cultural and interpersonal factors (i.e. partner interference) that physically or psychologically undermine a woman’s ability or motivation to adhere to ART.*

2a) Line 122: Did the authors alter the DHS questionnaire to focus on relationship-length violence? Since the DHS measures only past-year and lifetime, alterations must have been made to focus on violence ever being perpetrated by the current partner? If so, this is an interesting innovation and should be discussed further.

2b) The authors use self-reported lifetime IPV restricted to the current partner. Since they first write that they use the DHS IPV module and then (correctly) state that it measures IPV by current and former partners (page 6-7), the claim that the authors focus on lifetime IPV of the ongoing relationship is confusing. Either the claim is wrong or the authors have modified the DHS IPV module. If they have modified it, this should be clearly stated.

*Our study focused on relationship-specific IPV and we did this by recruiting only the women who were currently in an intimate partnership (either married or in a relationship). The DHS questions on IPV are framed as ‘Did your (last) husband/partner ever..’ to capture IPV by current or former partner. To make the questions fit to our focus, we removed the word ‘last’ so that our questions read ‘Did your husband/partner ever..’. We apologise for not clarifying this earlier and we have added a few lines in Pg. 7 from line 129 explaining this.*

3*)* Methods Note: The other aspects of the model are not discussed- why did the authors choose the predictor variables they did? What is the theory/conceptual model driving these models?

*Thank you for pointing this out. Within the data collection section, we have rewritten the sentence on the independent variables to state why they were used (Pg. 7 from line 146). We hope that this, together with the now added theoretical framework, will make it easier for the reader to understand why we included these covariates.*

4) I am concerned with the “woman is violent” covariate. This implies a bidirectionality of IPV that is not theoretically or conceptually consistent with violence in this context. I recommend removing it or strongly justifying it in the (yet nonexistent) paragraph explaining the other model covariates.

*Thank you for the comment. This covariate was added because it is an aspect which the DHS IPV questionnaire measures. Since we believe there was a hypothesis behind adding it in the domestic violence module, were interested in investigating whether it would influence our results. From the results, the variable does not add any strong argument for or against our hypothesis. It also does not stand out as a strong point of discussion. Nevertheless, for the sake of transparency we presented it because it was significant in the bivariate analyses and in some points of the logistic regression.*

5). Line 166: Can the authors provide a citation or reference to whether 100 shillings is standard compensation for such a survey? Non-Kenyan audiences may call into the question the coerciveness of this amount without this context.

*To the best of our knowledge there is no standard compensation for such a survey that we can reference. The research was paid for using limited private funds. After consulting with the AMPATH leadership and the healthcare workers, we agreed that KSh 100 (a little less than 1€ at the time) which was all we could afford, was better than giving nothing.*

*With regard to the possible coerciveness of this amount, we describe in the procedure section (Pg. 8 line 166 -174) that consent to participate in the study was sought through explaining the purpose of the study and reassuring the women that participation or non-participation was purely voluntary and would not affect their access to ART. The compensation was offered only after consent was given and the women had completed the questionnaire. Moreover, in terms of purchasing power KSh 100 is unfortunately very low. We would have wished to offer the participants more, had we had proper funding.*

6) Line 179: “virological” is not a word- should be “viral” suppression

*To the best of our knowledge, virological suppression and viral suppression can be used interchangeably. We also decided to use “virological suppression” as it was already used in other papers published in PlosOne and PlosMedicine, most recently Plymoth M. et al [**1**] and Hermans, L.E. et al [**2**].However, we are happy to use the term ‘viral suppression’ instead if it fits better to the discourse of PlosOne.*

7) Line 192: Despite the potential bias of small numbers of second-level units, the authors should have performed a sensitivity analysis to determine if results varied widely using a two-level model. The authors should have at least included clinic as a fixed effect at level 1 if not using multilevel modelling to control for the effect of sampling site

*Thank you for highlighting this. To provide the reader with the additional information we have added more details on the analyses we conducted before reaching the decision on our final model on Pg. 10 from line 200. That is, we decided against a multilevel modelling procedure due to the comparatively low intraclass correlation coefficient of ICC=.15 which we derived from an unconditional multilevel logistic regression model. Additionally, with as few as 12 clusters, fixed-effect estimates associated with level-2 predictors could have been severely biased. Adding clinic as an independent variable as part of our sensitivity analysis revealed differences among the clinics but there were no significant changes in the respective model parameter estimates from previous models.*

8) Since data on IPV during the last 12 months are available, which in my view would have been more suitable for the study than lifetime IPV, the authors should at least comment on how the results are altered if they are used.

*Noted. Within the data analysis section (Pg. 9 line 184), we have now added a short paragraph explaining that we also ran a second analysis considering only exposure to IPV in the last 12 months. The results of this second analysis have been added in the results section (Pg. 11 line 223-225, and Pg. 12 line 261-267). The findings mirror those of the relationship-specific IPV analysis; i.e. experiencing IPV reduces the odds of achieving optimal ART adherence, while education level and having an HIV-positive partner increased the odds of achieving optimal ART adherence. This further strengthens our rationale that specific IPV incidents may not affect medication adherence, but living in an environment in which IPV occurs does.*

9a) Line 210: I assume the authors did not try to fit logistic regression models with such small cell sizes as in Age<20 (n=1). Categories of the predictor variables should be reported in the way they were modelled in the final table.

9b) Line 228: Why are variables modelled differently in Table 1 vs Table 2 (e.g. Age modelled as continuous in table 2 but categorical in table 1?). These should be consistent.

9c) What does marital status, monogamy, polygamy in Table 1 mean? The classification is unclear

*Thank you for bringing these points to our attention. We apologise for the oversight and have changed them accordingly (Pg. 11)*

10) The authors write as if unadjusted results are reported, see p. 11 lin3-4. However, they are not reported.

*Thank you for pointing this out. We had presented both the unadjusted and adjusted results in our earlier manuscripts, hence the tone in reporting and the references. We have now revised this to fit to what is actually presented in the current manuscript (Pg. 12 from line 251).*

11) Line 214: I recommend using positive verbiage here- i.e. “physical, sexual, and any type of IPV remained significant”

*Noted. We changed the text following the reviewer’s suggestion (Pg. 12 line 252-253).*

12) Line 246: citations needed here for the Kenyan context

*We have added the relevant citations (Pg. 14. Line 289).*

13) Typo, p.19, line 6. on should be of.

*Noted. We decided to change the statement to ‘A review on programs in sub-Saharan Africa which have implemented IPV screening and counselling services in healthcare settings, reported that the interventions were positively received by both healthcare providers and clients’ (Pg. 19 line 412).*

14a) Line 372: I feel the conclusion needs work. What are the final lessons learned and next steps for this line of inquiry- additional thoughts from the researchers would be welcome here.

14b) “ART programs should consider incorporating basic IPV interventions into regular clinic services and using healthcare providers as information and navigation points for women exposed to IPV” does not follow from the findings. Such a conclusion should be based on evidence of a casual effect of IPV on ART adherence. An association does not have to be due to the impact of IPV on adherence. For example, women who end up with violent men might be different from others in ways not captured by the control variables, making them care less about their health. Moreover, violent men might have characteristics that influence their partners’ adherence. One example is that they are likely to be less faithful and less caring. Thus, a program that reduces IPV might not improve adherence. Although it seems reasonable that IPV actually has a causal effect on adherence, the conclusion drawn from the study should be modified.

*Noted. We decided to combine the two points because we believe they are related.*

*It is true that our findings show only a negative association and not a causal effect of IPV on ART adherence. From a researcher’s point of view, we consider this a limitation (as stated in the limitation section Pg. 18 line 389). However, from a practitioner’s point of view we still stand by our recommendation to consider introducing brief IPV screening and counselling at clinic level as a preventive measure. As existing literature on ART has established, the factors that influence ART adherence are diverse [**3-9**]. Therefore, it is true that in the end, any reduction in the effects of IPV may not result in improved ART adherence. Nonetheless, since our findings tell us that exposure to IPV could create a group that is vulnerable to suboptimal ART adherence, we recommend identifying these women who need extra monitoring and support. At the same time, given the high prevalence of IPV and knowing that, in general, IPV has negative effects on women’s health, an intervention would be good for the HIV-positive woman’s overall quality of life. As it stands, the jury is still out on whether IPV screening and counselling at clinical level has direct or indirect benefits on women’s health. We request future researcher to share their findings.*

*We have adjusted the future practices and conclusion section to reflect these sentiments (Pg. 19 line 398).*

15) A related issue is the consequences of IPV on HIV-infections. The authors describe these in the first two paragraphs of the paper and refer to several papers. However, they miss some important references and the fact that recent evidence indicates that there might not be a causal effect of IPV on HIV-infections among women in Sub-Saharan Africa. Women who live with violent men are primarily infected because violent men are more likely than others to be HIV-infected. See for example, Durevall and Lindskog (2015) and Heise and McGrory (2016).

*Thank you for the comment.*

*It is true that there are studies that question the bidirectional association between IPV and positive HIV serology. Although from our understanding, the two papers mentioned do not completely rule out the possibility of an association, but rather emphasise the importance of considering the woman’s partner’s sexual behaviour. That is, interventions should also focus on changing men’s sexual behaviour.*

*Nevertheless, it is true that we did not mention studies that counter the association between IPV and HIV infection. This is because our study focus was not HIV infection, but rather ART adherence. We therefore believe it is more relevant for us to discuss studies which support or reject that IPV has an influence on ART adherence. We understand that this might not have been clear before and we hope that including the theoretical framework helps to clarify it.*

16) A drawback of the study is that women who have not entered an ART program or have dropped out are not included in the sample. This should be made clear early on, particularly since it is likely to create a bias that weakens the association. It should also be commented on in Limitations. Now it is mentioned in Conclusion, which is odd.

*Noted. In our view, ‘not including women who have not entered the ART program in our sample’ is not a limitation because our aim was to determine IPV’s influence on ART adherence. Our target was therefore HIV-positive women who were already on ART. We agree that IPV could be a factor that prevents women from initiating ART (HIV care cascade step 3) and we have referenced, within the introduction section, some studies that highlight this (Pg. 4 from line 64).*

*With regard to not including women who have dropped out of the ART program, we have moved the mentioned drawback to the limitation section (Pg. 18 line 386). We have also retained it in the conclusion section because we believe it offers a point for future research.*

**References**

1. Plymoth M, Sanders EJ, Van Der Elst EM, Medstrand P, Tesfaye F, Winqvist N, et al. Socio-economic condition and lack of virological suppression among adults and adolescents receiving antiretroviral therapy in Ethiopia. PloS one. 2020;15(12):e0244066. PubMed PMID: 33320900. Pubmed Central PMCID: 7737988.

2. Hermans LE, Carmona S, Nijhuis M, Tempelman HA, Richman DD, Moorhouse M, et al. Virological suppression and clinical management in response to viremia in South African HIV treatment program: A multicenter cohort study. PLoS medicine. 2020 Feb;17(2):e1003037. PubMed PMID: 32097428. Pubmed Central PMCID: 7041795 Research and Development (ZonMW/WOTRO) during the study. AMJW further reports grants and consultancy fees from Janssen, Merck, ViiV Healthcare, and Gilead; grants and nonfinancial support from CLJI; and nonfinancial support and other from Virology Education, outside the submitted work, all paid to the University Medical Center Utrecht.

3. Ammassari A, Trotta MP, Murri R, Castelli F, Narciso P, Noto P, et al. Correlates and predictors of adherence to highly active antiretroviral therapy: overview of published literature. Journal of acquired immune deficiency syndromes. 2002 Dec 15;31 Suppl 3:S123-7. PubMed PMID: 12562034.

4. Cohen MH, Cook JA, Grey D, Young M, Hanau LH, Tien P, et al. Medically eligible women who do not use HAART: the importance of abuse, drug use, and race. American journal of public health. 2004 Jul;94(7):1147-51. PubMed PMID: 15226135. Pubmed Central PMCID: 1448413.

5. Nemes MI, Carvalho HB, Souza MF. Antiretroviral therapy adherence in Brazil. Aids. 2004 Jun;18 Suppl 3:S15-20. PubMed PMID: 15322479.

6. Lazo M, Gange SJ, Wilson TE, Anastos K, Ostrow DG, Witt MD, et al. Patterns and predictors of changes in adherence to highly active antiretroviral therapy: longitudinal study of men and women. Clinical infectious diseases : an official publication of the Infectious Diseases Society of America. 2007 Nov 15;45(10):1377-85. PubMed PMID: 17968839.

7. Hodgson I, Plummer ML, Konopka SN, Colvin CJ, Jonas E, Albertini J, et al. A systematic review of individual and contextual factors affecting ART initiation, adherence, and retention for HIV-Infected pregnant and postpartum women. PloS one. 2014 Nov 5;9(11). PubMed PMID: WOS:000344556900047.

8. Ayuo P, Musick B, Liu H, Braitstein P, Nyandiko W, Otieno-Nyunya B, et al. Frequency and factors associated with adherence to and completion of combination antiretroviral therapy for prevention of mother to child transmission in western Kenya. Journal of the International AIDS Society. 2013;16:17994. PubMed PMID: 23336727. Pubmed Central PMCID: 3536941.

9. Winchester MS. Synergistic vulnerabilities: Antiretroviral treatment among women in Uganda. Global public health. 2015 Aug 9;10(7):881-94. PubMed PMID: WOS:000359350300007.
