# Supplementary material for: Intimate partner violence is a barrier to antiretroviral therapy adherence among HIV-positive women: Evidence from government facilities in Kenya
Source: PLoS One. 2021 Apr 21;16(4):e0249813. doi: 10.1371/journal.pone.0249813 (PMC8059826; doi:10.1371/journal.pone.0249813)
Supplement: Attachment — Submitted filename: Response to Reviewers.docx [file pone.0249813.s007.docx]

**Reviewer’s comments**

**Reviewer #3**

The authors have responded well to many of the previous reviewers’ comments. I believe that the manuscript can be forwarded to the editor for final approval, provided some small changes are made. Most significant among these are the expansion of the conceptual model in the Introduction and the explanation of a few key findings in the Discussion.

Introduction:
• The conceptual model is still lacking in my opinion. There are three distinct ways in which IPV may lead to negative uptake of health services, including ART. I refer the authors to the seminal WHO conceptual model contained in the 2013 Global and regional estimates of violence against women: prevalence and health effects of intimate partner violence and non-partner sexual violence report.

*Thank you for highlighting this. We have decided to add a paragraph on Pg.5 from line 88. In this paragraph we describe the different pathways in which IPV directly and indirectly affects women’s health and how this could link to ART adherence. We have taken into account the abovementioned conceptual model.*

Methods:
• Although I personally disagree with the decision not to use multilevel modeling, the authors justify this decision well and there is no reason to suspect this decision meaningfully changed the results or their interpretation.

*Noted*

• I recommend moving the justification for using the stepwise regression approach to the Methods section from the Limitations section.

*Done. Please see Pg. 10 line 206*

Discussion:
• What are the authors’ hypotheses regarding the mechanisms or reasons behind why women who fought back and experience controlling behavior have reduced ART adherence? This should be discussed.

*Noted. Our results show that adjusting for controlling behaviour, women who fought back during an IPV encounter were more likely to report reduced odds of ART adherence than those who are never violent. Violence by women who are exposed to IPV is not uncommon as studies show it is one of the means through which some women defend themselves and their children [**1-3**]. Since we know that women who are exposed to IPV adopt different physical and nonphysical ways of protecting themselves, we cannot confidently draw any conclusions about the mentioned sub-group of women without having further assessments.*

*Our results suggest that the act of fighting back during an IPV encounter has no effect on ART adherence. This is evident by the fact that there is no effect when adjusted for physical and sexual IPV. However, a negative effect is seen when adjusted for controlling behaviour, which speaks more to the environment. We interpret this as being reflective of the underlying and underestimated detrimental effect controlling behaviour has, and encourage further research. One way would be through assessing the home environment in which the controlling behaviour occurs. Moreover, we believe that the negative effect on optimal adherence which is seen when adjusted for emotional IPV and controlling behaviour, furthers our rationale that it is not the IPV act itself, but rather living within an environment where IPV occurs, that affects a woman’s ability or motivation to adhere to ART.*

*We nevertheless realise our error in not making this clear in the beginning and have added a few lines explaining this in Pg 18 from line 343.*

**Reviewer #4**

The revised version of the paper is fine and I have only some very minor comments.

On line 106-108 the authors write that “We set a minimum duration of six months since beginning ART to recruit women who were accustomed to the ART routine, in an effort to reduce factors affecting adherence that may be related to the initiation stages of ART.” I would like to have some more arguments. Why would using a much smaller duration affect the results? There would be more noise, but you would have more observations and I assume that the correlation with IPV would not be affected. Did you test using a shorter duration?

*Thank you for this comment. We would like to address this in two ways. Firstly, we did not test using a shorter duration because it would have meant that we needed to also measure factors that are negatively associated with ART initiation. This includes but is not limited to: the woman’s knowledge and understanding of HIV/ART, the woman’s denial, fears or expectations of HIV/ART, challenges to the practical demands of treatment (e.g. scheduling, reluctance to start lifelong treatment), health worker-patient relations, adverse drug reaction, and regimen adjustments. Additionally, women who attend the ART program are referred through Home Based Counselling and Testing (HBCT), Voluntary Counselling and Testing (VCT) or Provider Initiated Testing and Counselling (PITC). This already puts them at different levels in terms of acceptance of HIV diagnosis, CD4 count levels, and their motivation to take up or adhere to treatment. All these together with the IPV related variables would inflate the number of independent variables we assess in our sample of 408 women.*

*Secondly, from a practitioner’s perspective, because of the aforementioned factors, we expect some instability in ART adherence in the first few months. According to the national guidelines for antiretroviral therapy in Kenya [**4**], it is after six months following initiation of ART that a patient is expected to have sufficient knowledge of HIV/ART and the importance of adherence, and is therefore expected to be compliant. Before six months, the guidelines recommend frequent appointments (two weeks, monthly) to assess proper administration and storage of the medication, to assess patient’s clinical progress, and to counsel on adherence. It is also during this duration that the patient is closely monitored for adverse drug reactions, drug resistance, and adjustments to the treatment regimen is made. At six months, a CD4 count is recommended as a follow up to the baseline CD4 count which was conducted when the HIV status was confirmed. We therefore expected that the women who participated in our survey had sufficient knowledge of HIV, medication compliance, and the importance and benefits of ART adherence.*

*The number of observations were not affected since we had no trouble achieving the target sample size using the inclusion criteria. Nonetheless, your comment has made us realise that this is information that may not be clear to the readers and so we have added a brief explanation on Pg. 6 from line 113.*

I do not understand this part. “We also did not include clinics as a fixed effect because this would lead to empty cells due to limited sample size. However, we included clinic as an independent variable as part of our sensitivity analysis” Fixed effects can be described as a dummy for each unit (clinic), which is what is used in the sensitivity analysis.

*Noted. We did not include clinics as a fixed effect in our main analysis because adding twelve additional dummy coded variables to the analysis would have led to predictor combinations with very few to zero observations. In this case, the specified model would have smoothed over the points where only limited data is available, which in turn would have led to a decrease in confidence in the respective models’ estimators. We have added this explanation on Pg 10 line 216.*

The authors have carried out estimations that are not reported, but they never state that they are not reported, such as the analysis of women experiencing IPV in the last 12 months. I think these results, and the sensitivity analysis, should be included in the on-line appendix, which now only contains one table.

*Noted. We have included the tables showing the results of the multiple regression models using only women who experienced IPV in the last 12 months, and the one including clinics is the Supplementary Information.*

The authors write that “However, this finding should be considered cautiously, since the tertiary education subgroup had only 49 respondents, which led to small cell numbers for some predictor combinations.” But 49 observations sound sufficient. And why would the cell for the tertiary education subgroup have fewer than 49 observations in some four reported regressions?

*Thank you for the comment. Out of all the women we randomly surveyed only 49 had tertiary education. When we combined education level with the other predictor variables there were instances where the cells were empty or had very few observations. For example, there were no women with tertiary education who were in a relationship and had experienced physical IPV. Only two women with tertiary education were in a polygamous marriage.*

*Nonetheless, we decided to rephrase the previous statement to read* ‘..*However, it should be noted that the tertiary education subgroup had only 49 respondents, which led to small cell or no numbers for some predictor combinations’ (Pg.18. line 339)*

In table 2, the p-value for Partner’s HIV status in model 3 and 4 are very different even though the estimates are very similar: 1.60 0.97 – 2.62 0.64; 1.60 0.98 – 2.63 .062. Typo?

*Thank you for bringing this to our attention. It was indeed a typo and we have corrected it.*

**References**

1. Swan SC, Snow DL. The development of a theory of women's use of violence in intimate relationships. Violence against women. 2006 Nov;12(11):1026-45. PubMed PMID: WOS:000241242700005. English.

2. Downs WR, Rindels B, Atkinson C. Women's use of physical and nonphysical self-defense strategies during incidents of partner violence. Violence against women. 2007 Jan;13(1):28-45. PubMed PMID: WOS:000242973100003. English.

3. Denson TF, O'Dean SM, Blake KR, Beames JR. Aggression in Women: Behavior, Brain and Hormones. Front Behav Neurosci. 2018 May 2;12. PubMed PMID: WOS:000431189600001. English.

4.-NASCOP. Guidelines for Antiretroviral Therapy in Kenya. Nairobi Kenya: National AIDS and STI Control Program, 2011.
